# New insights into constitutive neutrophil death

**DOI:** 10.1038/s41420-025-02287-1

**Published:** 2025-01-12

**Authors:** Tong Chen, Qian Ren, Fengxia Ma

**Affiliations:** 1 State Key Laboratory of Experimental Hematology, National Clinical Research Center for Blood Diseases, Haihe Laboratory of Cell Ecosystem, CAMS Key laboratory for prevention and control of hematological disease treatment related infection, Institute of Hematology & Blood Diseases Hospital, Chinese Academy of Medical Sciences &Peking Union Medical College, Tianjin, 300020 China; 2 Tianjin Institutes of Health Science, Tianjin, 301600 China

**Keywords:** Cell death, Innate immunity

## Abstract

Neutrophils undergo rapid aging and death known as constitutive or spontaneous death. Constitutive neutrophil death (CND) contributes to neutrophil homeostasis and inflammation resolution. CND has long been considered to be apoptotic until our findings reveal that it was a heterogeneous combination of diverse death. Furthermore, dead neutrophils retain functional roles via multiple manners. This review provides an overview of current research on the mechanism and modulation of CND. More noteworthy, we also summarize the after-death events of neutrophils. The fate of neutrophils can be changed under pathological conditions, so the involvement of CND in diseases and CND-related therapeutic strategies are also addressed.

## Facts

- Constitutive neutrophil death (CND) is a heterogeneous process that includes apoptosis and other forms of cell death, such as GSDME-mediated pyroptosis.
- Even after death, neutrophils retain physiological functions through events such as efferocytosis, extracellular vesicle generation, and plasma membrane rupture.
- CND is modifiable under pathological conditions and contributes to diseases such as inflammation, autoimmune disorders, and cancer, highlighting its potential as a therapeutic target.

## Open questions

- What molecular mechanisms drive the heterogeneity of CND? How are distinct modes of neutrophil death, such as apoptosis and pyroptosis, regulated and coordinated?
- Are there unexplored modes of CND or additional after-death functions yet to be identified?
- In what ways do diverse neutrophil fates influence disease progression and therapies?

## Introduction

Neutrophils make up the majority of white corpuscles in circulating blood, accounting for 50 ~ 70% in humans. As the first line immune responders against pathogenic invasion and other external stimuli, they are highly active and finely regulated in maintaining homeostasiss [1]. Every day, approximately one billion neutrophils per kilogram of adult body weight are generated from the bone marrow and undergo programmed cell death [2, 3]. Neutrophil death even in the absence of invasive infections or external triggers is characterized as constitutive neutrophil death (CND) or neutrophil spontaneous death. CND is essential for maintaining homeostasis of circulating neutrophils and inflammation resolution [4]. Accelerated neutrophil death decreases neutrophil amounts (neutropenia), heightening infection risk, whereas increased neutrophil numbers (neutrophilia) resulting from delayed neutrophil death are associated with chronic inflammation, autoimmune disorders, and cancer [4–7]. Under pathological conditions, neutrophils switch to specific modes of cell death (e.g., pyroptosis, necroptosis, ferroptosis, and NETosis) to participate in a variety of diseases (e.g., inflammation, autoimmune diseases, thrombosis, cancer) [1, 4, 8]. The mechanisms underlying CND provide valuable insights into intricate pathological processes and potential therapeutic strategies. In this review, we focus on the frontier of CND, outlining newfound mechanisms, with special attention to the heterogeneity of CND. Finally, the fate of neutrophils after CND is discussed as well.

## Advances in the mechanisms of constitutive neutrophil death

The conventional perspective posits that CND is apoptosis, characterized by classical morphological features, such as cell shrinkage, vacuolated cytoplasm, mitochondrial depolarization, nuclear condensation, DNA fragmentation and phosphatidylserine exposure on the cell membrane [4]. In a previous review, how various intracellular and extracellular modulators of neutrophil apoptosis has been outlined [4]. Nearly two decades of research have yielded novel insights.

### Reactive oxygen species

**Fig. 1:**
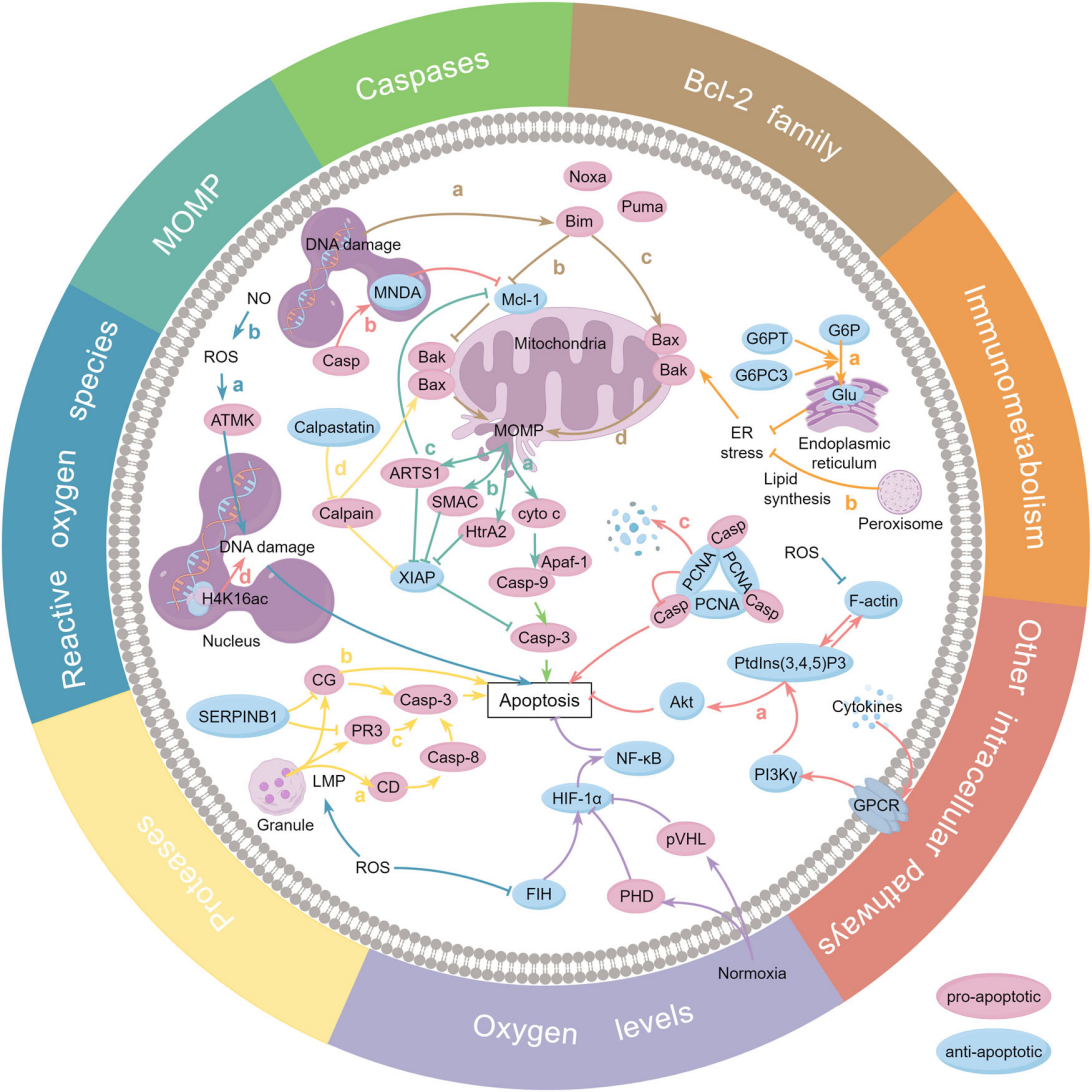
Mechanisms of constitutive neutrophil apoptosis. Neutrophils regulate their constitutive apoptosis through ROS generation (blue a-b), interaction network among Bcl-2 family proteins (taupe a-d), MOMP and mitochondrial proteins (cyan a-c), caspases cascade (green), LMP and proteases (jasmine a-d), immunometabolism (orange a-b), the HIF (lilac), and many other newly discovered intracellular signaling pathways (pink a-d). (By Figdraw: IOURRcd466). ROS reactive oxygen species, ATMK ataxia-telangiectasia mutated kinase, NO nitric oxide, H4K16ac histone H4 acetylation at lysine 16, Bim Bcl-2-interacting mediator of cell death, Noxa phorbol-12-myristate-13-acetateinduced protein 1, Puma Bcl-2-binding component 3, Mcl-1 myeloid leukemia cell differentiation protein Mcl-1, Bak Bcl-2 homologous antagonist/killer, Bax Bcl-2-associated X protein, MOMP mitochondrial outer membrane permeabilization, Casp caspase, MNDA myeloid nuclear differentiation antigen, cyto c cytochrome c, Apaf-1 apoptotic protease-activating factor 1, HtrA2 serine protease HTRA2, SMAC diablo IAP-binding mitochondrial protein, ARTS1 endoplasmic reticulum aminopeptidase 1, XIAP E3 ubiquitin-protein ligase XIAP, G6P glucose-6-phosphate, G6PT glucose-6-phosphate transporter, G6PC3 glucose-6-phosphatase-β, Glu glucose, ER endoplasmic reticulum, PCNA proliferating cell nuclear antigen, PtdIns(3,4,5)P3 phosphatidylinositol (3,4,5)-trisphosphate, PI3Kγ phosphoinositide 3-kinase γ, GPCR G-protein-coupled receptor, Akt protein kinase B, LMP lysosomal membrane permeabilization, CG cathepsin G, CD cathepsin D, PR3 proteinase 3, HIF hypoxia-inducible factor, FIH factor inhibiting HIF, PHD prolyl hydroxylases, pVHL von Hippel Lindau protein.

CND is known to be mediated by reactive oxygen species (ROS). Intriguingly, intracellular non-phagosomal-generated ROS, rather than ROS derived from phagosomes, initiates human neutrophil apoptosis, highlighting the importance of ROS origin over its production in driving neutrophil death [4, 9, 10]. Recently, a study reported that neutrophils from ataxia telangiectasia patients displayed a delayed CND. Oxidative burst activates ataxia-telangiectasia mutated (ATM) kinase, a modulator of DNA damage, to induce neutrophil death, thereby establishing DNA damage as a downstream mediator of ROS-driven neutrophil death [11] (Fig. 1 blue a). Honda F et al. identified Btk as a ROS-dependent gatekeeper of human neutrophil apoptosis. When tumor-necrosis factor (TNF) receptors or Toll-like receptors are engaged, Btk defective neutrophils generate more ROS that is linked to increased apoptosis and can be rescued by recombinant Btk transduction [12]. Nitric oxide (NO) synthesis by NO synthase (NOS) also modulates ROS level. The level of ROS, NO/inducible NOS (iNOS) increased with time in spontaneously dying human and mouse neutrophils. The pro-apoptotic role of NO/iNOS in CND was confirmed by the delayed neutrophil death from iNOS knockout (KO) mice. Mechanically, NO induces ROS production, caspase-8 and capase-3 cleavage, culminating in neutrophil apoptosis [13] (Fig. 1 blue b).

### Bcl-2 family proteins

The Bcl-2 family is a well-studied regulator of the intrinsic apoptosis pathway and mitochondrial outer membrane permeabilization (MOMP), functioning either as promoters or inhibitors of cell death. It is subdivided into three functional subtypes: initiators, guardians, and executioners.

(1) The initiators, also known as ‘BH3-only proteins’ (only Bid also has a BH4 domain), include pro-apoptotic members such as Bcl-2-associated agonist of cell death (Bad), Bcl-2-interacting killer (Bik), BH3-interacting domain death agonist (Bid), Bcl-2-interacting mediator of cell death (Bim), Bcl-2-modifying factor (Bmf), phorbol-12-myristate-13-acetate-induced protein 1 (Noxa), and Bcl-2-binding component 3 (Puma). They bind to executioners directly as activators or interact with guardians as sensitizers.

(2) Anti-apoptotic proteins such as Bcl-2, induced myeloid leukemia cell differentiation protein Mcl-1 (Mcl-1), Bcl-2-like protein 1 (Bcl-xL), Bcl-2-like protein 2 (Bcl-w), and Bcl-2-related protein A1 (A1) are classified as guardians that inhibit direct activators and executioners by binding to their BH3 domains.

(3) Bcl-2 homologous antagonist/killer (Bak), and Bcl-2-associated X protein (Bax) are considered to be pro-apoptotic executioners of MOMP [14].

There is consensus that CND is determined by the rivalry between pro-apoptotic and anti-apoptotic Bcl-2 family members [10]. Most Bcl-2 family proteins’ precise function in CND has been extensively investigated using KO mice (Supporting information, Table S1). Drawing from the available evidence, we propose an interaction network among Bcl-2 family members during CND (Fig. 1 taupe a-d). Bim serves as the main initiator of CND in response to upstream triggers such as DNA damage. Noxa and Puma work synergistically [15–17] (Fig. 1 taupe a). Mcl-1 is the master guardian in CND and decreases with CND. Bim, Noxa, and Puma interact with Mcl-1 to promote its proteolysis, thereby eliminating the sequestration of executioner Bax/Bak by Mcl-1 [16, 18, 19] (Fig. 1 taupe b). Alternatively, they may also activate Bax/Bak directly (Fig. 1 taupe c). They then oligomerize on the outer membrane of mitochondria to promote MOMP, accelerating neutrophil apoptosis [14] (Fig. 1 taupe d).

**Fig. 2:**
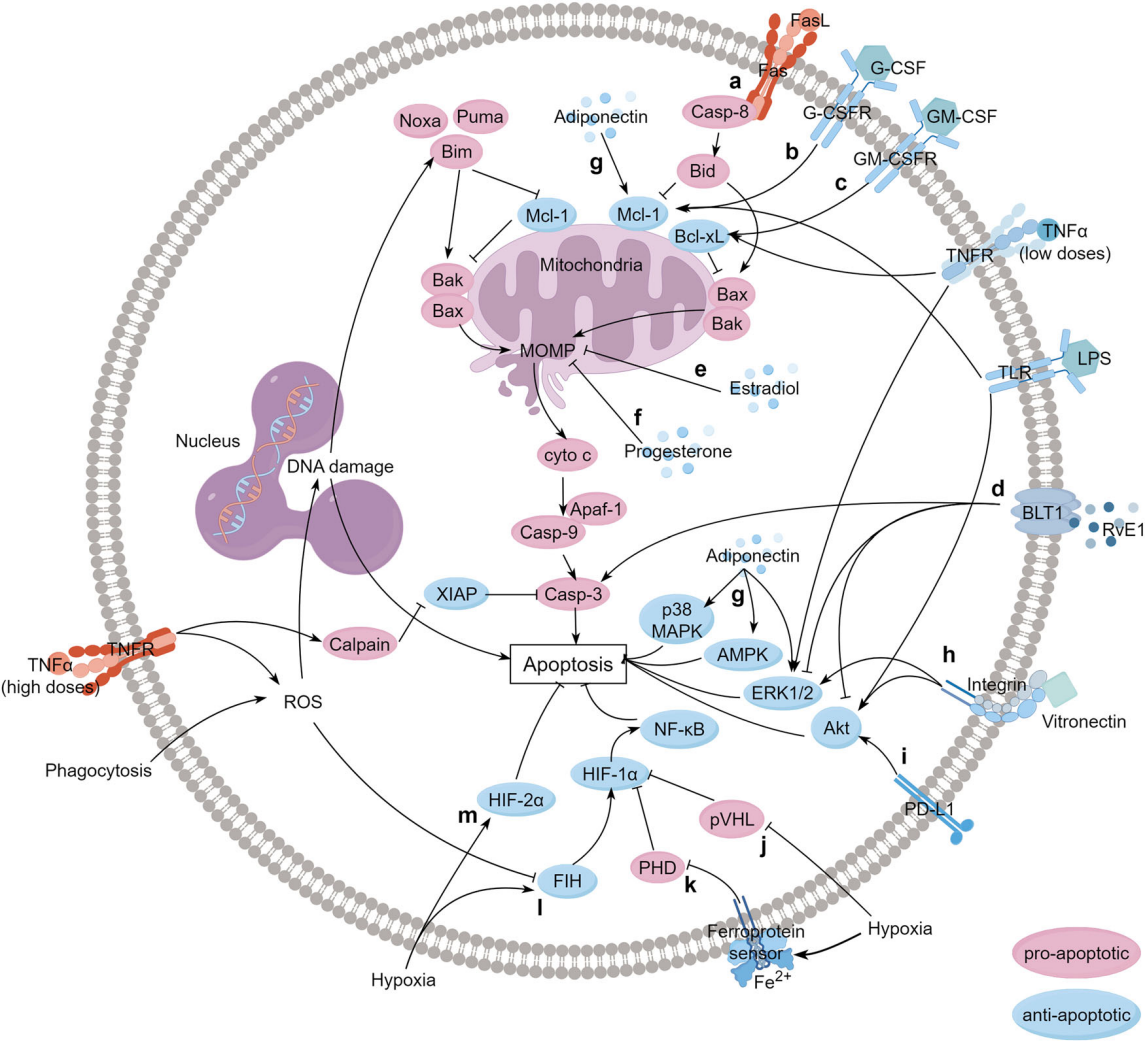
Extracellular modulators of neutrophil apoptosis. This figure focuses on recently published extrinsic modulators of neutrophil apoptosis. **a** Caspase-8-activated Bid and the executioner Bax/Bak promote Fas-induced neutrophil apoptosis. **b**–**c** G-CSF mainly targets Mcl-1 to postpone neutrophil apoptosis, but GM-CSF upregulated Bcl-xL to be the main guardian. **d** RvE1, as an endogenous lipid mediator, promotes ROS generation and apoptotic caspases activation, and simultaneously negatively regulates ERK- and Akt-mediated anti-apoptotic signals, thereby predisposing neutrophils to phagocytosis-induced apoptosis. **e**–**f** Physiological doses of estradiol and progesterone delay neutrophil apoptosis by reducing cyto *c* release and subsequent caspase activation. **g** Adiponectin reduces neutrophil apoptosis by stabilizing Mcl-1 and decreasing caspase-3 cleavage. It also activates MAPK, ERK 1/2, Akt, and AMPK signaling. **h** The interaction between vitronectin and β1/β3/β5 integrins activates Akt and ERK1/2 to diminish neutrophil apoptosis, independently of p38 signaling. **i** PD-L1 is upregulated in sepsis patients’ neutrophils and activates PI3K/Akt signaling to delay septic neutrophil death. **j–m** Hypoxia regulates HIF family proteins to delay neutrophil apoptosis. (By Figdraw: PPRSAd2e22). ROS reactive oxygen species, Bim Bcl-2-interacting mediator of cell death, Noxa phorbol-12-myristate-13-acetate-induced protein 1, Puma Bcl-2-binding component 3, Bid BH3-interacting domain death agonist, Mcl-1 myeloid leukemia cell differentiation protein Mcl-1, Bcl-xL Bcl-2-like protein 1, Bak Bcl-2 homologous antagonist/killer, Bax Bcl-2-associated X protein, MOMP mitochondrial outer membrane permeabilization, G-CSF granulocyte-colony stimulating factor, GM-CSF granulocytemacrophage colony-stimulating factor, XIAP E3 ubiquitin-protein ligase XIAP, RvE1 Resolvin E1, BLT1 leukotriene B4 receptor, MAPK mitogen-activated protein kinase, AMPK adenosine monophosphate-activated protein kinase, ERK extracellular signal-regulated kinase, Akt protein kinase B, HIF hypoxia-inducible factor, FIH factor inhibiting HIF, PHD prolyl hydroxylases, pVHL von Hippel Lindau protein.

Fas-deficient *lpr* mutant neutrophils die normally without stimulation, indicating that Fas-induced signaling is not required in CND [20]. Nonetheless, in response to pathogens, Fas induces caspase-8-activated Bid and subsequently Bax/Bak to promote neutrophil apoptosis [15–17] (Fig. 2a). In the pathological microenvironment, additional granulocyte-macrophage colony-stimulating factor (GM-CSF), and granulocyte-colony stimulating factor (G-CSF) also delay neutrophil death by fine-tuning the Bcl-2 family interaction network [18, 19, 21, 22] (Supporting information, Table S1) (Fig. 2b, c).

### MOMP and mitochondrial proteins

Executioners of the Bcl-2 family drive MOMP to liberate cytochrome *c* (cyto *c*). Apoptosome formation requires the assembly of cyto *c*, apoptotic protease-activating factor 1 (Apaf-1), and caspase-9. This complex is essential for initiating the downstream caspase cascade [4] (Fig. 1 cyan a). In human neutrophils, mitochondria play a minimal role in oxidative phosphorylation for adenosine triphosphate (ATP) synthesis, but retain their capacity to mediate cell death. The remarkably low levels of cyto *c* in neutrophils are both essential and sufficient for caspase activation. Other mitochondrial proteins, such as serine protease HTRA2 (HtrA2/Omi) and diablo IAP-binding mitochondrial protein (SMAC), are considered to play important roles in human neutrophil apoptosis as well [23, 24] (Fig. 1 cyan b). In addition, endoplasmic reticulum aminopeptidase 1 (ERAP1/ARTS1) is released from the mitochondrial membrane upon apoptotic stimuli, antagonizes E3 ubiquitin-protein ligase XIAP (XIAP) and mediates the degradation of Bcl-2 family guardians. ARTS, upstream of the mitochondria, triggers the caspase activation and subsequent apoptosis. Spontaneous death of neutrophils of Sept/ARTS^-/-^ mice is reduced, demonstrating the pro-apoptotic role of ARTS in CND [25] (Fig. 1 cyan c).

### Caspases

Caspases are evolutionarily conserved cysteine-dependent proteases that cleave substrates at specific aspartate residues. They are classified as either apoptotic or inflammatory based on their distinct functions and structures. Inflammatory caspases, such as caspase-1, -4, -5, and -11, share a caspase recruitment domain (CARD) at their N-terminal region. Apoptotic caspases comprise two functional subgroups: initiators (caspases-8, -9, and -10) and executioners (caspases-3, -6, and -7). Initiators activate executioners to trigger apoptosis. Notably, inflammatory and apoptotic caspases demonstrate functional overlap, with evidence of crosstalk between them [26].

Human neutrophils express a diverse range of caspases, including caspases-1, -3, -4, -6, -7, -8, -9, and -14 [10]. Over the years, many laboratories have utilized different inhibitors to investigate the role of caspases in CND. Hongbo R. Luo’s team discovered that pan-caspase or caspase-3-specific inhibitors dramatically delay human CND. Remarkably, blockage of the mitochondrial (caspase-9) or extrinsic (caspase-8) pathways fails to impede CND. It implied that caspase-3 acts as the primary effector caspase responsible for human CND [27]. Since whole-body KO of caspase-3, -7, -8, and -9 results in embryonic or perinatal lethality in mice, direct evidence from KO models is lacking [28]. To address this challenge, the use of neutrophil-specific KO mice is recommended.

### Proteases

Neutrophils are characterized by their abundant granules, which play a critical role in pathogen elimination through the release of cytotoxic compounds. Granule-released proteases are components of the apoptotic cascade [10]. Azurophilic granules release cathepsin D (CD) in a ROS–dependent way, which directly activates caspase-8, initiating neutrophil death during inflammation resolution (Fig. 1 jasmine a). CND is delayed by genetic deletion of CD but not cathepsin B in mice [29]. Cathepsin G (CG) is released via granule permeabilization. It promotes murine neutrophil apoptosis through caspase-independent and -dependent pathways (Fig. 1 jasmine b). Serpin family B member 1 (SERPINB1) protects neutrophils by inhibiting CG, which is verified by the fact that SERPINB1-deficient mice with bone marrow neutropenia reach complete remission by genetic deletion of CG, but not neutrophil elastase (NE) [30]. Hongbo R. Luo’s team found that proteinase 3 (PR3) released via lysosomal membrane permeabilization (LMP) during aging cleaves procaspase-3, eventually leading to both human and murine neutrophil apoptosis (Fig. 1 jasmine c). SERPINB1 also counteracts the pro-apoptotic effect of PR3 in senescent neutrophils [27].

Furthermore, the balance between the cytosolic protease calpain and its endogenous inhibitor calpastatin determines human neutrophil apoptosis. During neutrophil spontaneous death, calpastatin degradation results in increased calpain activity [31]. Calpains may cleave Bax and block XIAP to promote neutrophil apoptosis [4, 10] (Fig. 1 jasmine d). G-CSF inhibits calpains via control of Ca^2+^-influx, thereby increasing XIAP stability and inhibiting caspase-9 and caspase-3, ultimately delaying neutrophil death [32].

### Immunometabolism

Circulating neutrophils predominantly utilize glycolysis and the pentose phosphate pathway as their primary metabolic strategies, supporting their biological functions under both homeostatic and pathological conditions. Fatty acid oxidation, tricarboxylic acid (TCA) cycle, and oxidative phosphorylation are also essential for neutrophils in both steady and inflammatory states [33]. However, the relationship between CND and metabolic pathways is not well understood. Neutrophils express glucose-6-phosphate transporter (G6PT) and glucose-6-phosphatase-β (G6PC3) to transport glucose-6-phosphate (G6P) into the endoplasmic reticulum (ER) and hydrolyze it to glucose (Fig. 1 orange a). Neutrophils from G6PT-deficient mice display increased ER stress and apoptosis. Mechanistically, the intrinsic apoptosis pathway is activated [34]. In another study, murine G6PC3-deficient neutrophils also show accelerated apoptosis, which can be restored by G-CSF [35]. Hence, G6P translocation and glucose synthesis are necessary for a normal neutrophil lifespan. Similar to glucose metabolism disorders, impaired lipid synthesis selectively increases ER stress and neutrophil death by preferentially reducing peroxisome-derived membrane phospholipids containing ether bonds [36] (Fig. 1 orange b). In the TCA cycle, succinate is oxidized to fumarate by succinate dehydrogenase (SDH), which works as a ubiquinone oxidoreductase in respiratory chain complex II as well. Neutrophils from SDH-mutated patients display elevated intracellular succinate levels, reduced CND and improved hypoxic survival. This phenotype is associated with compromised complex II performance and decreased oxidative stress, independent of HIF-1α stabilization [37]. Thus, the participation of TCA cycle and respiratory chain in CND is uncovered. Sarah R. Walmsley’s team discover that neutrophils directly regulate mitochondrial function via glycerol 3-phosphate pathway in response to hypoxia, releasing mitochondrial ROS (mtROS) and maintaining polarized mitochondria. Through the suppression of prolyl hydroxylases (PHD), increased mtROS stabilizes hypoxia-inducible factor 1-α (HIF-1α), extending neutrophil lifespan within hypoxic circumstances [38]. In another case, caspase recruitment domain 9 (*Card9*) deletion in murine neutrophils remodels metabolism by reducing glycolysis and excessively activating mitochondria to a dysfunctional state, thereby generating mtROS that promotes neutrophil apoptosis, especially in oxidative stress. This explains the reduced viability of blood neutrophils from inflammatory bowel disease (IBD) patients with IBD-associated CARD9 single nucleotide variation [39]. The role of mtROS varies with cellular state and microenvironment. On the one hand, mtROS serves as a signaling molecule to help neutrophils adapt to hypoxia; on the other hand, it can also inflict damage and accelerate neutrophil death under specific conditions. This underscores an intricate role of mitochondrial metabolism in neutrophil death and the complicated regulation of intracellular ROS signaling.

In addition to intracellular energy metabolism, extracellular metabolites and hormones such as endogenous lipid mediator, estradiol, progesterone and adiponectin also regulate neutrophil death [40–43] (Fig. 2d–g).

### Other newly discovered intracellular signaling pathways

With the continuous discovery of various intracellular signaling pathways, our understanding of CND regulatory mechanisms has been greatly enriched. An early study by Hongbo R. Luo revealed that deactivation of phosphatidylinositol (3,4,5)-trisphosphate (PtdIns(3,4,5)P3)/Akt signaling results in human CND [44]. ROS accumulation in aging neutrophils inhibits the actin-mediated amplifying cycle to suppress PI3Kγ/PtdIns(3,4,5)P3/Akt signaling, driving CND [45] (Fig. 1 pink a). Myeloid nuclear differentiation antigen (MNDA), belonging to Pyrin and HIN domain (PYHIN) family, is caspases-cleaved and cytoplasm-accumulated, promoting Mcl-1 breakdown and fueling human neutrophil apoptosis. Impaired MNDA accumulation may contribute to prolonged neutrophil death in sepsis patients [46] (Fig. 1 pink b). Proliferating cell nuclear antigen (PCNA) was originally identified in proliferating cells’ nucleus as a DNA polymerase co-factor. Véronique Witko-Sarsat et al. uncovered human neutrophils expressed cytosolic PCNA that served as a protein interaction platform in a trimer form to sequester procaspases. In CND, PCNA homotrimers are modified by unknown mechanisms to facilitate PCNA proteasomal degradation. Its dissociation from procaspases allows the consequent caspase cascade activation, which ultimately boosts neutrophil apoptosis [47, 48] (Fig. 1 pink c). Cyclin-dependent kinase 7/9 mediated RNA polymerase II phosphorylation maintaining human neutrophil survival was demonstrated by treatment with their inhibitors [49]. The histone post-translational modifications in CND have been revealed recently. Histone H4 acetylation at lysine 16 is enriched at specific DNA regions of human peripheral neutrophils, preparing for chromatin breakage early in CND [50] (Fig. 1 pink d).

Recently, the intracellular mechanisms by which some cell surface molecules, such as integrin and PD-L1 delay neutrophil apoptosis have also been delineated [51, 52] (Fig. 2h, i).

### Oxygen levels

Tissue damage and inflammation shape a localized hypoxic microenvironment. Hypoxia may profoundly alter CND to affect disease progression and prognosis. Years of research by Sarah R. Walmsley and her collaborators have shown that hypoxia reversibly delays neutrophil apoptosis, and this is mediated by the transcription factor HIF-1α. Freshly isolated human neutrophils express HIF-1α mRNA, but HIF-1α protein is only detected in hypoxia. The von Hippel-Lindau (pVHL) protein interacts with HIF-1α and promotes its degradation under normal oxygen conditions. Hypoxia inhibits this interaction, allowing HIF-1α to accumulate and delay neutrophil apoptosis [53–55] (Fig. 2j). pVHL-mediated HIF-1α proteolysis requires the prolylhydroxylation of HIF-1α by PHD. Hypoxia stimulates a ferroprotein sensor that inhibits PHD, reducing HIF-1α degradation and consequently delaying CND [53] (Fig. 2k). In addition, an asparaginyl hydroxylase named factor inhibiting HIF (FIH) upregulates HIF-1α transcription in an oxygen-sensing way. It cooperates with PHD/pVHL to further contribute to HIF-1α accumulation and prolonged neutrophil lifespan in hypoxia [54, 56] (Fig. 2l). HIF-1α activates NF-κB to delay CND in hypoxia, independent of PI3K pathway [54]. A zebrafish inflammatory model confirms that HIF-1α stimulation reduces neutrophil death, delaying the resolution of inflammation [57]. Freshly isolated or normoxic human neutrophils express HIF-2α protein, which is further induced in hypoxia. HIF-2α is induced later than HIF-1α mainly during inflammation resolution to delay neutrophil apoptosis, leading to tissue damage (Fig. 2m). HIF-2α serves as another potential therapeutic target for chronic inflammation in addition to HIF-1α [58].

## Heterogeneity of constitutive neutrophil death

Through time-lapse microscopic video, we unexpectedly found that in addition to typical apoptosis, neutrophils underwent lytic cell death in CND, represented by AV/PI double-positive swelling cells. The swelling form coupled with early PI staining suggested disrupted membrane integrity, indicating lytic cell death [59]. Thus, we proposed heterogeneity of CND.

### GSDME-mediated pyroptosis in constitutive neutrophil death

Pyroptosis is a form of programmed cell death characterized by cell swelling, and plasma membrane disruption, resulting in massive leakage of cytoplasmic contents. The activation of inflammatory caspases (caspase-1/4/5/11) has long been considered a hallmark of pyroptosis [60]. Until 2015, gasdermin D (GSDMD) was identified as an executor of pyroptosis, downstream of inflammatory caspases [61–63]. GSDMD is cleaved by inflammatory caspases to produce N-terminal fragment (GSDMD-NT) and C-terminal product (GSDMD-CT). This cleavage abolishes the autoinhibition of GSDMD-NT by GSDMD-CT. GSDMD-NT oligomerizes to perforates the cell membrane, triggering pyroptosis and releasing inflammatory cytokines [64, 65]. GSDMD can be cleaved by NE, CG, and caspase-11 to be involved in neutrophil death [66–68]. Inflammasome activation in neutrophils cleaves GSDMD to promote IL-1β maturation and release without pyroptosis [69, 70]. Neutrophils generally resist GSDMD-mediated pyroptosis except under specific challenges such as *pseudomonas aeruginosa* [71].

In our recent work, GSDMD cleavage was observed during CND. Consistent with previous study [67], CND was retarded in GSDMD-deleted neutrophils. However, the proportion of lytic to apoptotic cells was unaltered in GSDMD-deleted neutrophils during spontaneous death. In summary, GSDMD globally accelerated CND without impact on the death mode [72].

**Fig. 3:**
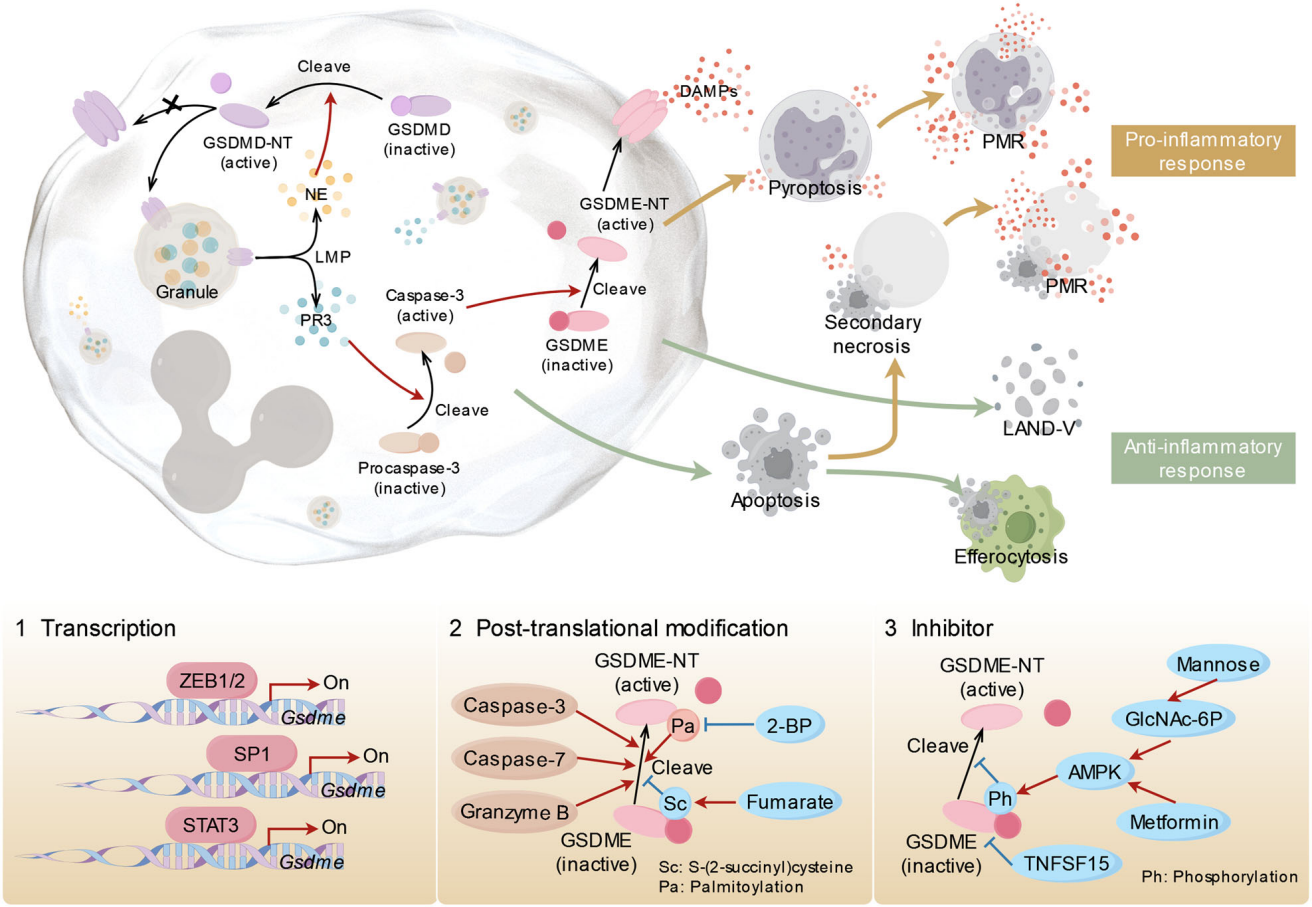
GSDME-mediated neutrophil pyroptosis and its potential regulatory factors. In aging neutrophils, proteinase 3 (PR3), released through lysosomal membrane permeabilization (LMP), cleaves procaspase-3. Activated caspase-3 then cleaves GSDME to trigger pyroptosis. GSDMD is also cleaved by neutrophil elastase (NE) or cathepsin G (CG) released by LMP, but instead of causing pyroptosis by forming pores on the cell membrane, GSDMD-NT perforates granules to amplify LMP. Different death modes and post-death fates of neutrophils determine inflammatory responses. Given that GSDME-mediated neutrophil pyroptosis seems to be a promising target, various regulatory factors at different levels are summarized. Of note, the above mechanisms are mainly studied in other cell types and remain to be verified in neutrophils. (By Figdraw: AAAYTbd5df). GSDMD gasdermin D, GSDME gasdermin E, LMP lysosomal membrane permeabilization, NE neutrophil elastase, PR3 proteinase 3, DAMPs damage-associated molecular patterns, PMR plasma membrane rupture, LAND-V large aging neutrophil-derived vesicles, SP1 specificity protein 1, STAT3 signal transducer and activator of transcription 3, 2- BP, 2-bromopalmitate Sc S-(2-succinyl)cysteine, Pa palmitoylation, GlcNAc-6P N-acetylglucosamine-6-phosphate, AMPK adenosine monophosphate-activated protein kinase, TNFSF15 tumor necrosis factor superfamily member 15, Ph phosphorylation.

Gasdermin E (GSDME, also called DFNA5) is another gasdermin family member. Granzyme B or caspase-3 cleave GSDME into GSDME-NT to form pores on cell membranes and induce pyroptosis in GSDME-expressing tumors [73, 74]. Upon infection with *Yersinia*, GSDME was activated in a RIPK kinase-dependent manner to drive murine neutrophil pyroptosis [75]. We found that GSDME was gradually activated downstream of the PR3/caspase-3 axis during CND. Interestingly, GSDME deficiency did not alter the overall death rate of murine neutrophils, but shifted lytic death entirely toward apoptosis, resulting in enhanced efferocytosis and accelerated inflammation resolution. In mouse models of bacterial pneumonia and acid aspiration pneumonia, neutrophil-specific GSDME deletion attenuated pulmonary damage and facilitated inflammation resolution, offering a possible therapeutic approach to limit inflammation by regulating neutrophil death modes [72]. From these discoveries, we propose more new insights into CND:

(1) Modulating cell death modes is an effective immune regulatory mechanism. Senescent and dead cells are eliminated in vivo by both lytic and apoptotic cell death, which cause pro- and anti-inflammatory reactions respectively. Neutrophils not only undergo apoptosis but also pyroptosis, which determines opposite immune responses. GSDME deletion does not affect the overall progression of CND, but skew their death from pyroptosis to apoptosis. Increased apoptotic neutrophils avoid inflammatory cytokines leakage and enhance efferocytosis by macrophages, thus augmenting anti-inflammatory responses. This anti-inflammatory mechanism worked in various murine models of sterile and bacterial pneumonia. Therefore, switching neutrophil death modes effectively regulates immune responses under both physiological and pathological conditions.

(2) GSDMD and GSDME have distinct and nonoverlapping functions in CND. GSDMD and GSDME are the two major gasdermin proteins expressed in neutrophils. GSDMD plays a well-established role in mediating pyroptosis in macrophages [61–63]. However, neutrophils seem far less susceptible to GSDMD-mediated pyroptosis than macrophages [69, 70]. Such resistance may be partially explained by SERPINB1 inhibiting serine proteases and inflammatory caspases in neutrophils [68, 76]. GSDMD is previously believed to contribute to neutrophil extracellular trap (NET) formation, but increasing evidence points to its dispensability [77–79]. In macrophages, GSDMD and GSDME share overlapping roles in pyroptosis. GSDME can compensate for GSDMD deficiency in pyroptosis execution [80]. In neutrophils, they function distinctly in CND. GSDMD regulates the overall rate of CND, while GSDME specifically mediates neutrophil pyroptosis and its loss skews death to apoptosis [72].

(3) Distinct activation mechanisms drive GSDMD and GSDME to function differently in CND (Fig. 3). In aging neutrophils, LMP-mediated PR3 release initiates caspase-3 activation, which then cleaves GSDME to trigger pyroptosis. GSDMD is cleaved by NE or CG released by LMP [67, 68], but GSDMD-NT perforates granules to amplify LMP rather than oligomerizing on cell membrane to mediate pyroptosis [70]. However, whether GSDME has effect on the LMP positive feedback loop is unknown.

(4) GSDME, as a physiological pyroptosis executor, offer a promising therapeutic target. Its druggability in tumor and other immune cells has been well documented in previous studies [73, 74, 81, 82]. Our work highlights its role in resolving inflammation as a checkpoint for neutrophil spontaneous pyroptosis.

Given that GSDME-mediated neutrophil pyroptosis seems to be a promising target, we summarize various regulatory factors of GSDME (Fig. 3). (1) Transcription: Epithelial-mesenchymal transition-activating transcription factors ZEB1/2 directly drives transcriptional activation of GSDME in human cancer [83]. Specificity protein 1 (Sp1) interacts directly with the GSDME promoter to boost its transcription in Hela cells [84]. GSDME expression is also positively regulated by signal transducer and activator of transcription 3 (STAT3) in mouse peritoneal macrophages [85]. (2) Post-translational modification: GSDME is cleaved at Asp-270 by caspase-3 or granzyme B to relieve autoinhibition and initiate pyroptosis in human cancer [73, 74]. GSDME reacts with fumarate to form S-(2-succinyl)cysteine that limits its cleavage by caspases and ability to initiate macrophage pyroptosis [86]. GSDME palmitoylation on C-terminal facilitate its separation from GSDME-NT, augmenting chemotherapy drug-induced pyroptosis, which could be inhibited by the palmitoylation inhibitor 2-bromopalmitate [87]. (3) Inhibitor: In retinal cells, tumor necrosis factor superfamily member 15 (TNFSF15) prevents high glucose-triggered pyroptosis by interacting with GSDME [88]. Increased mannose metabolism and N-acetylglucosamine-6-phosphate (GlcNAc-6P) levels activate AMPK, then phosphorylates GSDME to suppress pyroptosis in both cancer and normal cells [89]. Of note, the above mechanisms are mainly studied in cell types other than neutrophils and remain to be verified in neutrophils. The regulation of GSDME in neutrophils is an uncharted territory waiting to be explored.

## After-death events of neutrophils

Although the mode and mechanism of CND are thoroughly studied, the events after neutrophil death are rarely known. A little evidence demonstrates that death is not the end of neutrophils, and that dead neutrophils prolong their functionality via multiple manners.

Clearance of apoptotic cells is critical for tissue homeostasis, as uncleared corpses can undergo secondary necrosis, intensifying inflammation and triggering autoimmunity [90]. The process of apoptotic cell engulfment, termed efferocytosis, is performed by both non-professional phagocytes (e.g., endothelial and epithelial cells) and professional phagocytes (e.g., macrophages and dendritic cells) [91]. The anti-inflammatory effect of efferocytosis is mediated by macrophages secreting anti-inflammatory cytokines including IL-10, transforming growth factor (TGF-β), and platelet-activating factor (PAF) and subsequently inhibiting proinflammatory cytokines [92]. Resolution phase macrophage-derived IFN-β was then identified as an effector cytokine in resolving bacterial inflammation by promoting neutrophil apoptosis and efferocytosis [93]. Pro-resolving lipid mediator alterations during efferocytosis have also been well profiled [94]. In 2005, Stark et al. demonstrated that phagocytosis of apoptotic neutrophils not only removed cellular remnants but also regulated neutrophil production in vivo via IL-17/IL-23-dependent G-CSF generation [95]. More interestingly, apoptotic neutrophils are dynamic entities instead of inert corpses awaiting removal. They release epidermal growth factor (EGF) and promote monocyte differentiation into tissue-resident antigen-presenting cells for activation of T cell effector functions [96]. According to Wang et al., apoptotic neutrophils-derived lysophosphatidylserine activates type 3 innate lymphoid cells to guide epithelial healing [97].

During the terminal stages of apoptosis, apoptotic cells can generate an array of apoptotic extracellular vesicle (EV) [98], allowing efficient cell clearance and carrying biomolecules for cell-to-cell communication [99]. Dieker and colleagues found that 15% of Annexin V ^+^ EVs originated from neutrophils in SLE patients, suggesting the existence of neutrophil-derived apoptotic EVs in vivo [100]. Shen GF et al. showed that EVs from apoptotic human neutrophils, but not the apoptotic neutrophils themselves, selectively downregulated the proliferation of CD25^neg^ CD127^pos^ Th cells by suppressing IL-2/IL-2R signaling [101].

Considerable understanding has been obtained in recent years in after-death events of apoptotic neutrophils. In contrast, events after neutrophil lytic death remain poorly defined. Plasma membrane rupture (PMR) is the final cataclysmic event in lytic cell death, causing complete cell lysis and the release of lactate dehydrogenase (LDH) and other damage-associated molecular patterns (DAMPs), which subsequently drive excessive inflammation and tissue injury. PMR was thought to be a passive event following cell death. However, a recent study suggested that was not the case. A pioneering study showed that the nerve injury-induced protein 1 (NINJ1) played an essential role in the induction of PMR. *Ninj1* deficient macrophages exhibited impaired PMR with a persistent ballooned morphology and failed to release LDH and HMGB1. In macrophage, PMR also occurred after apoptosis, necroptosis, and even bacterial pore-forming toxin-triggered cell death. Subsequently, Dondelinger Y et al. identified NINJ1 as a crucial regulator of plasma membrane permeabilization and consequent DAMP release during ferroptosis, parthanatos, H_2_O_2_-induced necrosis and secondary necrosis [102]. Degen M et al. showed that, during lytic cell death, the extracellular α-helices of NINJ1 inserted into the plasma membrane to polymerize NINJ1 monomers into amphipathic filaments that ruptured the plasma membrane [103]. During neutrophil spontaneous death, PMR was also observed. Our data by single-cell RNA sequencing showed that NINJ1 was highly expressed in neutrophils, especially in aging neutrophils. Further investigation is needed to determine whether NINJ1 mediates PMR during neutrophil spontaneous death and to elucidate its role in neutrophil-driven inflammation and tissue damage.

## CND alterations in diseases and therapeutic strategies

The rate and pattern of neutrophil death undergo dramatic alterations under disease conditions, profoundly influencing disease progression and therapeutic outcomes. During sepsis, constitutive neutrophil apoptosis is hindered, and neutrophils may undergo various other forms of cell death such as necrosis, necroptosis, pyroptosis, NETosis, and autophagy. The exact role and mechanisms of neutrophil death in sepsis are not yet entirely understood, presenting a significant challenge for future research [104]. Activated PD-L1/PI3K/Akt signaling and impaired cytoplasmic MNDA accumulation may partially account for delayed neutrophil death in sepsis patients [46, 52]. In systemic lupus erythematosus (SLE), FcγR activation downregulates SERPINB1 in neutrophils, leading to spontaneous activation of caspase-1/-11 and GSDMD cleavage. Simultaneously, mitochondrial DNA oxidation promotes GSDMD-NT oligomerization and cell death [105]. In addition, neutrophils in SLE patients are tilted toward ferroptosis because autoantibodies and interferon-α in the serum enhances the binding of the transcriptional repressor CREMα to the glutathione peroxidase 4 (the key ferroptosis regulator) promoter [106]. Neutrophils are increasingly recognized as participants in tumor immune responses. Lai Guan Ng and colleagues found a significant extension of the life span of the tumor neutrophils (135 hours) compared with peripheral blood neutrophils (91.5 hours). The tumor microenvironment drives the reprogramming of immature and mature neutrophils to localize to glycolytic and hypoxic niches and exert pro-angiogenic effects that favors tumor growth [107].

The aforementioned studies have employed pharmacological or genetic regulation of death-related signaling to improve disease outcomes, demonstrating numerous potential therapeutic targets. We summarize two main therapeutic strategies.

(1) Direct regulation of neutrophil death in vivo. First, regulating the rate of neutrophil death represents an effective approach. Adriano G Rossi et al. used the CDK inhibitor R-roscovitine to accelerate the resolution of neutrophilic inflammation in acute pleurisy, lung injury, and arthritis in mice [108]. Atiqur Rahma et al. identified ErbB signaling as a target that accelerated neutrophil apoptosis. The ErbB inhibitors gefitinib, CP-724714, erbstatin, tyrphostin AG825 successfully accelerated the resolution of neutrophilic inflammation in multiple inflammation models [109]. In contrast, the GSDMD inhibitor disulfiram significantly reduces SLE severity in mice by inhibiting neutrophil death [105]. More importantly, mediators that are redundant in CND but modulate neutrophil apoptosis under pathological conditions provide optimal targets, allowing selective depletion of pathological neutrophils, with minimal harm to homeostatic circulating neutrophils. For instance, Bcl-xL is functionally redundant in CND, but neutrophils switch to Bcl-xL for survival upon exposure to GM-CSF in inflammation or tumors. The selective Bcl-xL inhibitor A-1331852 has been shown to treat inflammation and tumors without causing neutropenia by reversing decelerated neutrophil apoptosis in relevant disease models [21, 22]. Numerous selective inhibitors targeting the Bcl-2 family have been developed, with some already in clinical application [110].

Second, switching neutrophil death patterns is another approach. We have demonstrated that genetic deletion of GSDME in neutrophils abolishes its pyroptosis to alleviate inflammation, but pharmacological inhibition of GSDME in neutrophils remains to be developed [72]. Ferroptosis inhibitors also alleviate SLE by inhibiting neutrophil ferroptosis in lupus-prone mice [106].

Finally, direct elimination of extracellular pathological niche is equally effective. The oxygen self-supporting CaO_2_ nanocoating on the implant surface destroys the hypoxic environment, accelerates neutrophil apoptosis, preventing implant-related infection [111].

(2) Transfusion with vitro trained neutrophils. Human neutrophils are inapplicable to genetic manipulation and transfusion therapy because of their limited lifespan after isolation. Anoxia combined with glucose supplementation that maintains viability and function of neutrophils for 20 hours provides new opportunities [112]. Through pharmacological screening, Hongbo R. Luo’s team developed a combined regimen, caspases–lysosomal membrane permeabilization–oxidant–necroptosis inhibition plus G-CSF (CLON-G). CLON-G potently extends human and murine neutrophil half-life from less than one day to more than five days with their critical functions preserved. Transfusion with CLON-G-treated neutrophils is proved to be safe and effective in murine models of neutropenia-associated bacterial pneumonia and systemic candidiasis [113]. Adoptive cell transfer therapy with pharmacologically treated or genetically edited functional neutrophils, though still distant from clinical application, presents fascinating prospects. In addition, further studies are needed to explore the therapeutic potential of ex vivo treated neutrophils under other pathological conditions.

## Conclusions and Future Perspectives

Recent studies suggest a complicated network of intracellular signaling pathways governing CND. The events after neutrophil death are tightly regulated as well. Although our understanding of CND has been greatly enriched, some fundamental questions remain to be answered. The initial events triggering CND has long been unresolved. Increasing evidence highlights the involvement of LMP. GSDMD-mediated LMP positive feedback loop activates downstream caspase cascades to drive global neutrophil death. During CND, GSDMD tends to oligomerize on organelle membrane, especially granule membrane, rather than forming holes on the plasma membrane [70]. The structural basis and biochemical mechanisms underpinning this phenomenon warrant further investigation. In addition to p30 GSDMD-NT, several studies have identified some non-classical cleavage fragments (p45 GSDMD, p13 GSDMD, and p23 GSDMD) and their non-pyroptotic functions [114–116]. Recently, ROS-dependent S-palmitoylation was found to determine the membrane translocation and pore-forming activities of GSDMD-NT and even full-length GSDMD [117–119]. Considering the functional differences of GSDMD in macrophages and neutrophils, it is thought-provoking whether these non-classical cleavage fragments and post-translational modifications of GSDMD exist and function during CND. Regarding GSDME, the executor of neutrophil spontaneous pyroptosis, its cleavage mechanism during CND has not been fully clarified. Although we show that GSDME is cleaved by caspase-3, caspase-3 inhibitor cannot totally block GSDME cleavage. Furthermore, whether other types of cell death such as ferroptosis, cuproptosis, disulfidptosis occur during CND is unknown [106, 120, 121]. A deeper understanding of the modes and molecular mechanisms driving CND is essential for identifying novel therapeutic targets and devising promising regimens for disorders linked to disrupted neutrophil death regulation, including infectious diseases, autoimmune disorders, and cancer. Likewise, since after-death events of neutrophils including efferocytosis, EV, PMR are crucial for inflammation and tissue damage, investigating the mechanisms and regulators of these processes appears highly worthwhile. In addition, cytokines, oxygen levels, metabolites, and intercellular communication in the microenvironment reprogram neutrophil death. CND variations in pathological niches of various diseases, as well as from peripheral blood to tissues, need to be characterized to identify more therapeutic targets. Last but not the least, the species differences between humans and experimental animals cannot be ignored. Therefore, in terms of experimental models, rare samples from gene-defective patients and in vitro-differentiated neutrophils derived from human stem cells, which are amenable to genetic manipulation, are anticipated to help confirm species similarities.

## Supplementary information

Table S1 The role of Bcl-2 family proteins in neutrophil apoptosis
