# Supplementary material for: New insights into constitutive neutrophil death
Source: Cell Death Discov. 2025 Jan 12;11:6. doi: 10.1038/s41420-025-02287-1 (PMC11725587; doi:10.1038/s41420-025-02287-1)
Supplement: Supplementary file 1 — Table S1 The role of Bcl-2 family proteins in neutrophil apoptosis [file 41420_2025_2287_MOESM1_ESM.docx]

Table S1. The role of Bcl-2 family proteins in neutrophil apoptosis.

| Name | Expression | Role | Evidence |
| --- | --- | --- | --- |
| Bcl-2 | **Human**  **RT-PCR:** Bcl-2 was not detected in freshly isolated human neutrophils, nor in human neutrophils during spontaneous death, GM-CSF delayed death, and TNF-α induced death^1^.  **RPA:** Faint Bcl-2 mRNA was detected in human neutrophils during spontaneous death. Treatment with GM-CSF/LPS/TNF-α/IFN-γ did not induce its expression^2^.  **WB:** Bcl-2 protein was absent from human neutrophils during spontaneous death. Treatment with GM-CSF/sodium butyrate/IL-1β/LPS did not induce its expression^2,3^.  **Mouse**  **WB:** Bcl-2 reduced continuously in Hoxb8 neutrophils during differentiation and spontaneous death^4^. | **1** Bcl-2 is absent from human neutrophils during constitutive death.  **2** Bcl-2 plays a redundant role in constitutive neutrophil apoptosis and Fas-induced neutrophil apoptosis in mice. | **1.1** Bcl-2 is undetectable in human neutrophils either at the mRNA level or at the protein level^1-3^.  **2.1** Neutrophils from peritoneal cavity (PC) and bone marrow (BM) of transgenic mice overexpressing Bcl-2 demonstrated markedly delayed spontaneous death^4-6^.  However, PC and BM neutrophils of bcl-2^-/-^ mice did not differ from wild-type (WT) neutrophils in spontaneous death^6^.  **2.2** Inhibition of Bcl-2 with ABT-737 markedly accelerated the death of FasL-treated hMRP8-Bcl-2 transgenic neutrophils but had no effect in WT neutrophils, which are more dependent upon Mcl-1^7^. |
| Mcl-1 | **Human**  **RPA:** Mcl-1 was one of the major Bcl-2 family transcripts found in human neutrophils. GM-CSF/LPS increased Mcl-1 mRNA, while TNF-α (10 ng/mL) decreased it^2^.  **WB:** Mcl-1 was expressed abundantly and declined during human neutrophil spontaneous death. GM-CSF greatly decreased the loss of Mcl-1^2,3^.  **Mouse**  **WB:** Mcl-1 reduced continuously in Hoxb8 neutrophils during differentiation and spontaneous death^4^.  Mcl-1 was easily detected and gradually decreased in primary mouse BM neutrophils during spontaneous death. GM-CSF/G-CSF/LPS increased its expression^4,8,9^. | **1** Mcl-1 is anti-apoptotic, the continuous reduction of which due to proteolysis serves as one of the main contributors to constitutive neutrophil apoptosis.  **2** Mcl-1 performs anti-apoptotic function in Fas-mediated neutrophil apoptosis.  **3** Mcl-1 is the main target of G-CSF to delay neutrophil apoptosis.  **4** Up-regulated Bcl-xL may be the dominant mediator of GM-CSF-delayed neutrophil apoptosis, while Mcl-1 acts as an alternative bypass, and A1 may also play a minor role. | **1.1** Neutrophils were generally reduced in the peripheral blood (PB), spleen, and BM of LysM-cre(+)/Mcl-1^Δ/Δ^ mice, and exhibited an enhanced spontaneous apoptotic rate than control cells^10^.  **1.2** Mcl-1 knockdown accelerated the spontaneous death of mature Hoxb8 neutrophils^9^.  **1.3** Mcl-1^+/–^ neutrophils had a significant survival disadvantage during spontaneous death compared to their control counterparts^11^.  **2.1** Vav-Mcl-1 transgenic neutrophils showed delayed responses to FasL stimulation^7^.  **2.2** Mcl-1^+/–^ neutrophils displayed more rapid Fas-induced apoptosis than WT ones^11^.  **3.1** Mcl-1^-/-^ neutrophils were completely resistant to the pro-survival effects of G-CSF^10^.  **4.1** GM-CSF significantly increased the level of MCL-1, BCL-XL, and A1 in neutrophils^8^.  **4.2** Mcl-1 knockout had little effect on GM-CSF delaying neutrophil apoptosis^10^.  **4.3** In the presence of ABT-737 (Bcl-2/Bcl-xL/Bcl-w inhibitor), knockdown of Mcl-1 in Hoxb8 neutrophils drastically abolished the anti-apoptotic effect of GM-CSF^9^.  **4.4** The Bcl-xL inhibitor (A-1331852) impaired the anti-apoptotic effect of GM-CSF in mouse neutrophils, while the Mcl-1 inhibitor (S63845) did not^8^.  **4.5** The Bcl-xL inhibitor (A-1331852) greatly eliminated the anti-apoptotic effect of tumor-derived supernatant on mouse BM neutrophils. GM-CSF was previously confirmed to be the main substance in the supernatant that delays neutrophil death^12^. |
| Bfl-1  (A1) | **Human**  **RPA:** A1 was one of the main transcripts detected in human neutrophils undergoing spontaneous death (3h). Stimulation with GM-CSF (50 ng/mL), LPS (100 ng/mL), TNF-α (10 ng/mL), or IFN-γ (100 U/mL) for 3 hours up-regulated A1 mRNA^2^.  **WB:** A1 protein could be detected in primary human neutrophils. It was strongly induced by GM-CSF, and LPS^9^.  **Mouse**  **WB:** The weak A1 protein expression was seen in primary mouse BM neutrophils and Hoxb8 neutrophils undergoing spontaneous death. GM-CSF but not G-CSF induced A1 expression ^8,9^. However, A1 has also been reported not to be detected in primary mouse BM neutrophils and Hoxb8 neutrophils, which may be due to different exposure time, antibody quality, or cell status^4,8^. | **1** A1 is important for survival in the neutrophil lineage especially early in differentiation.  **2** The three subtypes of A1 (a/b/d) may functions differently in constitutive neutrophil apoptosis, with A1-a tending to be anti-apoptotic.  **3** A1 may acts as an alternative mediator of GM-CSF-delayed neutrophil death, which can be fully compensated by Bcl-xL.  **4** A1 is functionally redundant in Fas-mediated or LPS-delayed neutrophil death, but promotes neutrophil survival after LPS plus GM-CSF stimulation. | **1.1** When the effect of A1 knockdown was uncovered by ABT-737 at the beginning of differentiation, significantly enhanced cell death was observed in A1-knonkdown Hoxb8 neutrophils^9^.  **2.1** Spontaneous death of A1-a^-/-^mice PB neutrophils was accelerated compared to WT mice or A1-a^+/-^ mice^13^.  **2.2** There was no protection by A1 overexpression in Hoxb8 neutrophils. The same was seen when A1 was overexpressed in Bim/Puma double-deficient neutrophils. Moreover, analysis of protein levels validated that overexpressed A1 was seen in both progenitors and differentiated neutrophils^4^.  **2.3** A1 konckdown appears to slightly promoted spontaneous death of Hoxb8 neutrophil^9^.  **2.4** There was no difference in neutrophil spontaneous death between A1^-/-^ mice and WT mice^11^.  **3.1** A1 deletion alone did not affect GM-CSF to delay neutrophil death, but exerted a negative impact when Bcl-xL was inhibited by A-1331852^8,11^.  **3.2** In the presence of ABT-737 (Bcl-2/Bcl-xL/Bcl-w inhibitor), knockdown of A1 in Hoxb8 neutrophils partly abolished the anti-apoptotic effect of GM-CSF^9^.  **4.1** A1 deletion did not affect LPS to delay neutrophil death^11^.  **4.2** A1 deletion did not affect FasL to induce neutrophil death^11^.  **4.3** A1 deletion impaired LPS plus GM-CSF to delay neutrophil death^11^. |
| Bcl-xL | **Human**  **RT-PCR/RPA:** Bcl-xL mRNA was detected in human neutrophils, the level of which was not modified by stimulation with GM-CSF (50 ng/mL), LPS (100 ng/mL), TNF-α (10 ng/mL), or IFN-γ (100 U/mL) for 3 hours^1,2^.  **WB:** Bcl-xL decreased with the spontaneous death of human neutrophils, which was blocked by caspase-3 inhibitor Z-DEVD-FMK. GM-CSF (100 U/mL), and LPS (10 ng/mL) did not change its level, but TNF-α (300 U/mL) facilitated its turnover. However, some studies speculated that Bcl-xL only detected at long exposures may come from contaminating PBMCs^1-3^.  **Mouse**  **WB:** Bcl-xL was maintained during differentiation and down-regulated during spontaneous death in Hoxb8 neutrophils ^4^. Bcl-xL was also found in primary mouse BM neutrophils, which could be up-regulated by GM-CSF, and G-CSF. Additionally, in murine models of arthritis, gout, and airway inflammation, the neutrophils at the site of inflammation expressed up-regulated Bcl-xL compared with PB neutrophils^8,12^.  **Flow cytometry:** Bcl-xL was expressed in all neutrophil populations from the BM, PB, lung, or tumors of healthy or tumor-bearing mice. The levels were elevated in healthy lung neutrophils and the highest in tumor-associated neutrophils^12^. | **1** Bcl-xL does not determine constitutive neutrophil death at homeostasis. Nevertheless, neutrophils switch to Bcl-xL for survival upon exposure to GM-CSF at the site of inflammation, and tumors. | **1.1** Bcl-xL inhibitor A-1331852 restored neutrophil apoptosis under inflammatory conditions ex vivo. Moreover, in murine models of acute and chronic inflammatory disease, Bcl-xL blockade reduced inflammatory neutrophil numbers and ameliorated tissue pathology. In contrast, there was minimal effect on circulating neutrophils^8^.  **Cell:**  mouce primary unsorted blood leukocytes  **GM-CSF:**  recombinant murine GM-CSF  10/100/1000 pg/mL  **Inhibitor:**  A-1331852 (inhibits Bcl-xL)  S63845 (inhibits Mcl-1)  ABT-199 (inhibits Bcl-2)  1 μM  added 16h after GM-CSF stimulation start **Time point:**  16h after inhibitor treatment start  **Apoptosis assay:**  propidium iodide  APC calibration beads  flow cytometry  **Result:**  A-1331852 partly eliminated the pro-survival effect of GM-CSF.  S63845/ABT-199 had no effect.  **1.2** Tumor cell-derived GM-CSF triggers the expression of Bcl-xL protein and enhances neutrophil survival through JAK/STAT signaling. Bcl-xL inhibitor A-1331852 blocked the induced neutrophil survival without impacting their normal lifespan in vitro and in vivo^12^.  **Cell:**  mouse primary BM neutrophils  **GM-CSF:**  tumor-derived supernatant, in which GM-CSF was confirmed to be the main substance that delays neutrophil death.  **Inhibitor:**  Venetoclax (inhibits Bcl-2)  Navitoclax (inhibits Bcl-2/Bcl-xL)  A-1331852 (inhibits Bcl-xL)  0.1/10/100 nM  added simultaneously with the supernatant  **Time point:**  24h after culture start  **Apoptosis assay:**  annexin V + 7-AAD  flow cytometry  **Result:**  A-1331852 partly eliminated the pro-survival effect of GM-CSF.  Navitoclax was less effective.  Venetoclax had no effect.  **1.3** ABT-737 did not compromise GM-CSF-mediated survival when added to the culture either simultaneously or 4 h after stimulation star, indicating that GM-CSF-enhanced survival is independent of Bcl-2, Bcl-xL or Bcl-w, but most likely entirely dependent on Mcl-1 and A1^9^.  **Cell:**  differentiated Hoxb8 neutrophils  **GM-CSF:**  B16 cell-derived 1% GM-CSF supernatant  recombinant murine GM-CSF (10 ng/mL)  **Inhibitor:**  ABT-737 (inhibits Bcl-2/Bcl-xL/Bcl-w)  1.5 μM  added simultaneously or 4h after GM-CSF stimulation start  **Time point:**  24/48/72h after culture start  **Apoptosis assay:**  live/dead fixable far red dead cell stain (molecular probes)  flow cytometry  **Result:**  ABT-737 had no effect. |
| Bcl-w | **Human**  **RPA:** Bcl-w was not detected in human neutrophils^2^.  **Mouse**  **WB:** Bcl-w was down-regulated during differentiation in Hoxb8 neutrophils^4^. | **1** Bcl-w is absent from mature neutrophils and has a redundant role in constitutive neutrophil death and Fas-mediated neutrophil apoptosis. | **1.1** BM and PC neutrophils from Bcl-w^-/-^ mice died at a normal rate in either constitutive death or FasL-induced apoptosis^6^. |
| Bax/ Bak | **Human**  **RT-PCR/RPA:** Bax and Bak were both expressed in human neutrophils^1,2^.  **WB:** The expression and variation of Bax and Bak in human neutrophils undergoing constitutive death and inflammatory cytokine-stimulated death remains controversial^1-3^.  **Mouse**  **WB:** Bax and Bak was up-regulated during differentiation in Hoxb8 neutrophils, while during constitutive death, Bax declined and Bak ascended ^4^. Apoptotic neutrophils possessed reduced levels of serine-phosphorylated Bax correlating with an increase in activated Bax as well as an increase in the amount of Bax found translocated to the mitochondria, which could be blocked by GM-CSF^14^. | **1** Bax alone may be redundant in constitutive neutrophil death and Fas-mediated neutrophil apoptosis.  **2** Bax/Bak-dependent activation of the intrinsic apoptotic pathway is required for efficient induction of apoptosis by FasL in neutrophils.  **3** Bax and Bak, as pro-apoptotic factors, mediate constitutive neutrophil death. | **1.1** BM and PC (elicited by 0.5% casein in 2mL PBS for 3h) neutrophils from Bax^-/-^ mice died at a normal rate in either constitutive or FasL-induced apoptosis. Survival was determined after 24/48/72 hours (BM neutrophils) or after 8/16/24/36 hours (PC neutrophils) by staining with annexin V plus propidium iodide and flow cytometric analysis^6^.  **1.2** PC (elicited by thioglycolate) neutrophils from Bax^-/-^ mice displayed resistance to spontaneous and TNF-α-induced apoptosis. Apoptosis was assessed by morphology after 12 hours^14^.  **2.1** Upon treatment with FasL, Bax^−/−^Bak^−/−^ neutrophils were less sensitive than WT neutrophils and died more slowly, but still more rapidly than the saline-treated controls^7^.  **3.1** The number of BM myeloid cells, and PB neutrophils increased in Bax^−/−^Bak^−/−^ mice^15^.  **3.2** Bax^−/−^Bak^−/−^ neutrophils were greatly resistant to constitutive apoptosis^8^. |
| Bad | **Human**  **RPA/WB:** Bad mRNA and protein was expressed stably in human neutrophils undergoing spontaneous death. GM-CSF (50 ng/mL 3h), LPS (100 ng/mL 3h), and TNF-α (10 ng/mL 3h) up-regulated Bad mRNA without changing protein levels, suggesting that the increase in mRNA was the result of accumulation due to enhanced stability rather than increased transcription^2^.  **RT-PCR/WB:** Levels of Bad mRNA decreased following GM-CSF (10 ng/mL) stimulation at 4 hours. GM-CSF substantially increased Bad phosphorylation at Ser112 and Ser136 and increased the cytosolic accumulation of Bad from membranes. TNF-α (200 U/mL) caused a biphasic effect on the rate of morphologic apoptosis, which corresponded to an early increase (4h), and a late inhibition (20h), of Bad mRNA levels^16^.  **Mouse**  **RT-PCR/WB:** The levels of Bad mRNA and protein remained constant in mouse BM/PC neutrophils dying spontaneously^6^. | **1** The role of Bad in neutrophil apoptosis remains unconfirmed due to lack of evidence from Bad knockout mice. |  |
| Bid | **Human**  **WB:** Bid decreased with human neutrophil spontaneous apoptosis^2^.  **Mouse**  **RT-PCR/WB:** Bid mRNA and protein remained constant with mouse neutrophil spontaneous apoptosis. During Hoxb8 neutrophil differentiation, Bid protein decreased^4,6^. | **1** Bid functions redundantly in constitutive neutrophil death.  **2** Bid is pro-apoptotic in Fas-mediated neutrophil apoptosis. | **1.1** BM neutrophils from Bid^-/-^ mice died spontaneously at a normal rate^4,11^.  **2.1** Upon treatment with FasL, Bid^−/−^ neutrophils were less sensitive and died more slowly than WT neutrophils^7,11^. |
| Bim | **Mouse**  **RT-PCR:** Bim mRNA appeared to be increased under conditions of spontaneous apoptosis in PC (elicited by 0.5% casein for 3h) but not in BM neutrophils^6^.  **WB:** Bim was increased during Hoxb8 neutrophil differentiation, whereas the levels of Bim in primary or Hoxb8 neutrophils remained invariable in spontaneous death^4,6^. | **1** Bim is pro-apoptotic in constitutive neutrophil death but not essential in Fas-mediated apoptosis. | **1.1** Bim deficiency delayed neutrophil spontaneous death, but did not affect FasL-induced apoptosis^4,6^. |
| Noxa | **Mouse**  **RT-PCR:** Noxa mRNA was unchanged with neutrophil spontaneous death^6^.  **WB:** Noxa was detected in progenitor cells and decreased during Noxb8 neutrophil differentiation, but not detected in mature neutrophils, possibly because Noxa binds to Mcl-1 and degrades with it^4,6^. | **1** Noxa, along with Puma, synergizes with Bim to trigger neutrophil spontaneous death. Mechanistically, Noxa may bind to Mcl-1 to induce its degradation, which then promotes spontaneous neutrophil death. | **1.1** Rate of neutrophil spontaneous death (slow to fast): Bcl-2-tg < Bim^-/-^Noxa^-/-^ < Bim^-/-^ Puma^-/-^ < Bim^-/-^ < Noxa^-/-^ ≈ Puma^-/-^ ≤ WT^4^. |
| Puma | **Mouse**  **RT-PCR:** Puma mRNA kept unchanged with neutrophil spontaneous death^6^.  WB: Puma increased first and then decreased during Noxb8 neutrophil differentiation, but not detected in mature neutrophils^4,6^. | **1** Puma, along with Noxa, synergizes with Bim to trigger neutrophil spontaneous death. | **1.1** Rate of neutrophil spontaneous death (slow to fast): Bcl-2-tg < Bim^-/-^Noxa^-/-^ < Bim^-/-^ Puma^-/-^ < Bim^-/-^ < Noxa^-/-^ ≈ Puma^-/-^ ≤ WT^4^. |
| Bik | **Human**  **RPA/WB:** Both Bik mRNA and protein were detected in human neutrophils, and the level of protein did not change with spontaneous death^2^. | **1** The role of Bik in neutrophil apoptosis remains unknown due to lack of evidence from Bik knockout mice. |  |
| Bmf | **Mouse**  **RT-PCR/WB:** Bmf protein increased but mRNA was consistent with neutrophil spontaneous death, which may be due to the enhanced protein stability caused by post-translational modifications^6^. | **1** The role of Bmf in neutrophil spontaneous death remains unknown. Bmf accumulation is granulocyte-specific and is not merely a consequence of cell death. | **1.1** Accumulation of Bmf was also observed in neutrophils from Bim^-/-^ or Bcl-2 transgenic mice. Bmf protein levels did not increase in thymocytes undergoing cytokine withdrawal–induced death^6^. |

**References**

1 Weinmann, P., Gaehtgens, P. & Walzog, B. Bcl-Xl- and Bax-alpha-mediated regulation of apoptosis of human neutrophils via caspase-3. *Blood* **93**, 3106-3115 (1999).

2 Moulding, D. A., Akgul, C., Derouet, M., White, M. R. & Edwards, S. W. BCL-2 family expression in human neutrophils during delayed and accelerated apoptosis. *J Leukoc Biol* **70**, 783-792 (2001).

3 Moulding, D. A., Quayle, J. A., Hart, C. A. & Edwards, S. W. Mcl-1 expression in human neutrophils: regulation by cytokines and correlation with cell survival. *Blood* **92**, 2495-2502 (1998).

4 Kirschnek, S. *et al.* Molecular analysis of neutrophil spontaneous apoptosis reveals a strong role for the pro-apoptotic BH3-only protein Noxa. *Cell Death Differ* **18**, 1805-1814 (2011). <https://doi.org:10.1038/cdd.2011.69>

5 Lagasse, E. & Weissman, I. L. bcl-2 inhibits apoptosis of neutrophils but not their engulfment by macrophages. *J Exp Med* **179**, 1047-1052 (1994). <https://doi.org:10.1084/jem.179.3.1047>

6 Villunger, A., Scott, C., Bouillet, P. & Strasser, A. Essential role for the BH3-only protein Bim but redundant roles for Bax, Bcl-2, and Bcl-w in the control of granulocyte survival. *Blood* **101**, 2393-2400 (2003). <https://doi.org:10.1182/blood-2002-07-2132>

7 Croker, B. A. *et al.* Fas-mediated neutrophil apoptosis is accelerated by Bid, Bak, and Bax and inhibited by Bcl-2 and Mcl-1. *Proceedings of the National Academy of Sciences of the United States of America* **108**, 13135-13140 (2011). <https://doi.org:10.1073/pnas.1110358108>

8 Carrington, E. M. *et al.* BCL-XL antagonism selectively reduces neutrophil life span within inflamed tissues without causing neutropenia. *Blood Adv* **5**, 2550-2562 (2021). <https://doi.org:10.1182/bloodadvances.2020004139>

9 Vier, J., Groth, M., Sochalska, M. & Kirschnek, S. The anti-apoptotic Bcl-2 family protein A1/Bfl-1 regulates neutrophil survival and homeostasis and is controlled via PI3K and JAK/STAT signaling. *Cell Death Dis* **7**, e2103 (2016). <https://doi.org:10.1038/cddis.2016.23>

10 Dzhagalov, I., St John, A. & He, Y. W. The antiapoptotic protein Mcl-1 is essential for the survival of neutrophils but not macrophages. *Blood* **109**, 1620-1626 (2007). <https://doi.org:10.1182/blood-2006-03-013771>

11 Schenk, R. L. *et al.* The pro-survival Bcl-2 family member A1 delays spontaneous and FAS ligand-induced apoptosis of activated neutrophils. *Cell Death Dis* **11**, 474 (2020). <https://doi.org:10.1038/s41419-020-2676-9>

12 Bodac, A. *et al.* Bcl-xL targeting eliminates ageing tumor-promoting neutrophils and inhibits lung tumor growth. *EMBO Mol Med* **16**, 158-184 (2024). <https://doi.org:10.1038/s44321-023-00013-x>

13 Hamasaki, A. *et al.* Accelerated neutrophil apoptosis in mice lacking A1-a, a subtype of the bcl-2-related A1 gene. *J Exp Med* **188**, 1985-1992 (1998). <https://doi.org:10.1084/jem.188.11.1985>

14 Gardai, S. J. *et al.* Phosphorylation of Bax Ser184 by Akt regulates its activity and apoptosis in neutrophils. *J Biol Chem* **279**, 21085-21095 (2004). <https://doi.org:10.1074/jbc.M400063200>

15 Lindsten, T. *et al.* The combined functions of proapoptotic Bcl-2 family members bak and bax are essential for normal development of multiple tissues. *Mol Cell* **6**, 1389-1399 (2000). <https://doi.org:10.1016/s1097-2765(00)00136-2>

16 Cowburn, A. S., Cadwallader, K. A., Reed, B. J., Farahi, N. & Chilvers, E. R. Role of PI3-kinase-dependent Bad phosphorylation and altered transcription in cytokine-mediated neutrophil survival. *Blood* **100**, 2607-2616 (2002). <https://doi.org:10.1182/blood-2001-11-0122>
